# Linking Dynamic Habitat Selection with Wading Bird Foraging Distributions across Resource Gradients

**DOI:** 10.1371/journal.pone.0128182

**Published:** 2015-06-24

**Authors:** James M. Beerens, Erik G. Noonburg, Dale E. Gawlik

**Affiliations:** 1 Department of Biological Sciences, Florida Atlantic University, Boca Raton, Florida, United States of America; 2 US Geological Survey, Southeast Ecological Science Center, Fort Lauderdale, Florida, United States of America; Central South University, CHINA

## Abstract

Species distribution models (SDM) link species occurrence with a suite of environmental predictors and provide an estimate of habitat quality when the variable set captures the biological requirements of the species. SDMs are inherently more complex when they include components of a species’ ecology such as conspecific attraction and behavioral flexibility to exploit resources that vary across time and space. Wading birds are highly mobile, demonstrate flexible habitat selection, and respond quickly to changes in habitat quality; thus serving as important indicator species for wetland systems. We developed a spatio-temporal, multi-SDM framework using Great Egret (*Ardea alba*), White Ibis (*Eudocimus albus*), and Wood Stork (*Mycteria Americana*) distributions over a decadal gradient of environmental conditions to predict species-specific abundance across space and locations used on the landscape over time. In models of temporal dynamics, species demonstrated conditional preferences for resources based on resource levels linked to differing temporal scales. Wading bird abundance was highest when prey production from optimal periods of inundation was concentrated in shallow depths. Similar responses were observed in models predicting locations used over time, accounting for spatial autocorrelation. Species clustered in response to differing habitat conditions, indicating that social attraction can co-vary with foraging strategy, water-level changes, and habitat quality. This modeling framework can be applied to evaluate the multi-annual resource pulses occurring in real-time, climate change scenarios, or restorative hydrological regimes by tracking changing seasonal and annual distribution and abundance of high quality foraging patches.

## Introduction

Species distribution models (SDM) link species occurrence with a suite of environmental predictors and have a wide range of applications in wildlife science and management by predicting species distributions across landscapes. They provide a powerful tool for land managers when the variable set captures the biological requirements of the species and can be manipulated to account for various management activities [1]. The resulting suitability metrics are most relevant to population dynamics in anthropogenically disturbed ecosystems when linked with measures of reproductive success and applied to a suite of restoration alternatives [2].

The efficacy of a SDM is impacted by the availability and choice of predictors and scale, the modeling method, and the degree of spatial and temporal extrapolation [3]. Moreover, many issues remain relatively unexplored in the SDM field concerning how a species’ ecology can affect model building and evaluation such as factors of conspecific attraction, phenotypic and behavioral plasticity, and response to environmental gradients. Recent reviews suggest that ecological theory is rarely considered in SDMs [3–5]. Habitat preference, for example, has in large part been assumed to remain unaltered as a function of the variety of habitat types available. However, animals are often confronted with an environment in which the types of habitats available at any given time are constantly changing. For species adapted to these systems, habitat selection has been shown to vary with stronger selection for resources that are relatively rare in the environment [5].

To address dynamic habitat selection, discrete-choice resource selection function (RSF) models were developed to model a series of choices with a discrete set of resources over time [6]. A weakness of this approach is that static resource selection models are still produced and only reflect average preference within the range of habitat conditions encountered during the study period [7]. Therefore, an important consideration must be given to modeling habitat selection over a wide-range of habitat availabilities in order to quantify the functional response in habitat selection [8]. A non-linear response will result when decreased availability of a resource in the landscape results in increased selection for another, which may be expected when, e.g., multiple limiting resources have been depleted by human activities [8–10].

While some authors have attempted to capture changing preference in response to the change in available resources [10–11], these measures did not reflect the changing area of habitat that may be available. Further, studies have commonly examined an animal’s selection of food resources over a spatial gradient of exposure to predators or anthropogenic disturbance [10,12]; however, in pulsed ecosystems foragers may exhibit dynamic responses to variation in food resources that arises from processes operating at multiple temporal scales. For example, in some aquatic ecosystems, an animal’s access to the prey base (i.e., prey availability) can fluctuate under seasonal cycles of drying and flooding [13–15]. In these habitats prey production requires multi-annual periods of inundation, whereas prey concentration at densities profitable for predators occurs over the shorter period of drying [13,16–17]. When wet season conditions result in low prey production, mobile predator species have shown selection for habitat in which the short-term drying process mitigates the loss of productive foraging habitat [17]. In this case, selection increases for one process when another is limiting, however the benefits of this behavior are unknown. These two processes, prey production and prey concentration, can also converge to create high quality habitat depending on climatic and hydrologic conditions. Thus, animals also depend on the habitat characteristics at multiple spatial scales, from individual patches to their distribution across the landscape.

The Florida Everglades is a dynamic subtropical wetland subject to seasonal resource pulses. A decline in the productivity generated by these pulses, across all trophic levels, resulted from water management practices and loss of spatial extent [18–19]. The desired condition of Everglades’ restoration is a hydrologic regime that recovers and sustains some of the defining ecological characteristics of the historic Everglades ecosystem [20]. Principally, historical hydrology has been associated with an abundant and stable population of wading birds, important indicator species for the Greater Everglades ecosystem [14]. Because wading birds are highly mobile top predators, their populations integrate productivity across trophic levels and over a large landscape scale. However, understanding and predicting wading bird responses to environmental restoration are hindered by their changing preference for hydrological characteristics depending on the production and concentration of prey, which occurs over different temporal scales. Multiple hydrological variables influence habitat selection and conspecific attraction that can fluctuate in strength depending on resource availability and foraging strategy of wading birds [17,21–22]. Human development at the wetland margins and ponding caused by water control structures now restrict the availability of high quality patches to a narrow range of hydrological conditions [13,23]. Further, changes in timing of favorable conditions have altered pre-breeding physiology [24], nesting initiations (i.e., phenology; [25]), chick provisioning [26], and nesting success [26]. An abundant prey base that is made available by receding water is required to support a large breeding population of wading birds through the end of the dry season when chicks fledge.

Additionally, increasing evidence suggests that changes in long-term habitat quality and prey availability have disparately affected wading bird species with a more constrained foraging niche (i.e., specialists; [17,24]. Across the Everglades, populations of wading bird species that require higher prey concentration, such as tactile foragers (i.e., White Ibis [*Eudocimus albus*] and Wood Stork [*Mycteria americana*]; hereafter ibises and storks), have disproportionally decreased from the 1930s to 2001 when compared with populations of visual foragers that favor relatively deeper water and have lower giving-up-densities of prey ([13]; i.e., Great Egret [*Ardea alba*]; hereafter egrets). This pattern likely indicates an overall decline in prey availability [14]. In addition, the ibis and stork, while similar in foraging strategy, differ in other traits such as prey size selection, foraging flight distance, nest initiation date, and nest cycle length [27] and thus may serve unique functions as indicators.

Here, we developed a modeling framework to predict habitat selection and abundance of three representative species of wading birds (egrets, ibises, and storks) using long-term, spatially explicit observational records combined with high temporal-resolution environmental predictors. This approach served three purposes: (1) to capture changing preference of wading birds across resource gradients, (2) to determine the effect of resource gradients on conspecific attraction, and (3) to evaluate species abundance in terms of the ecological trade-offs associated with the quantity, timing, and distribution of water. Furthermore, models included measures to account for both spatial autocorrelation and the area of available habitat. Using a multi-model approach, we aggregated wading bird foraging distributions over 1) space (temporal model) and 2) time (spatial model) to reduce the noise associated with individual locations and isolate variables representing processes that operate at different spatial and temporal scales. A spatial foraging conditions model (SFC) examined spatial and hydrological dynamics at a fixed spatial scale (i.e., cell) to predict wading bird frequency of use over time, whereas a temporal foraging conditions model (TFC) predicted flock and individual abundance across the landscape from daily habitat selection influenced by hydrological processes. The two models can be used to derive quantitative management indices for variation in patch quality (TFC) and patch abundance (SFC) over the landscape.

## Methods

### Data and Variables

**Fig 1:**
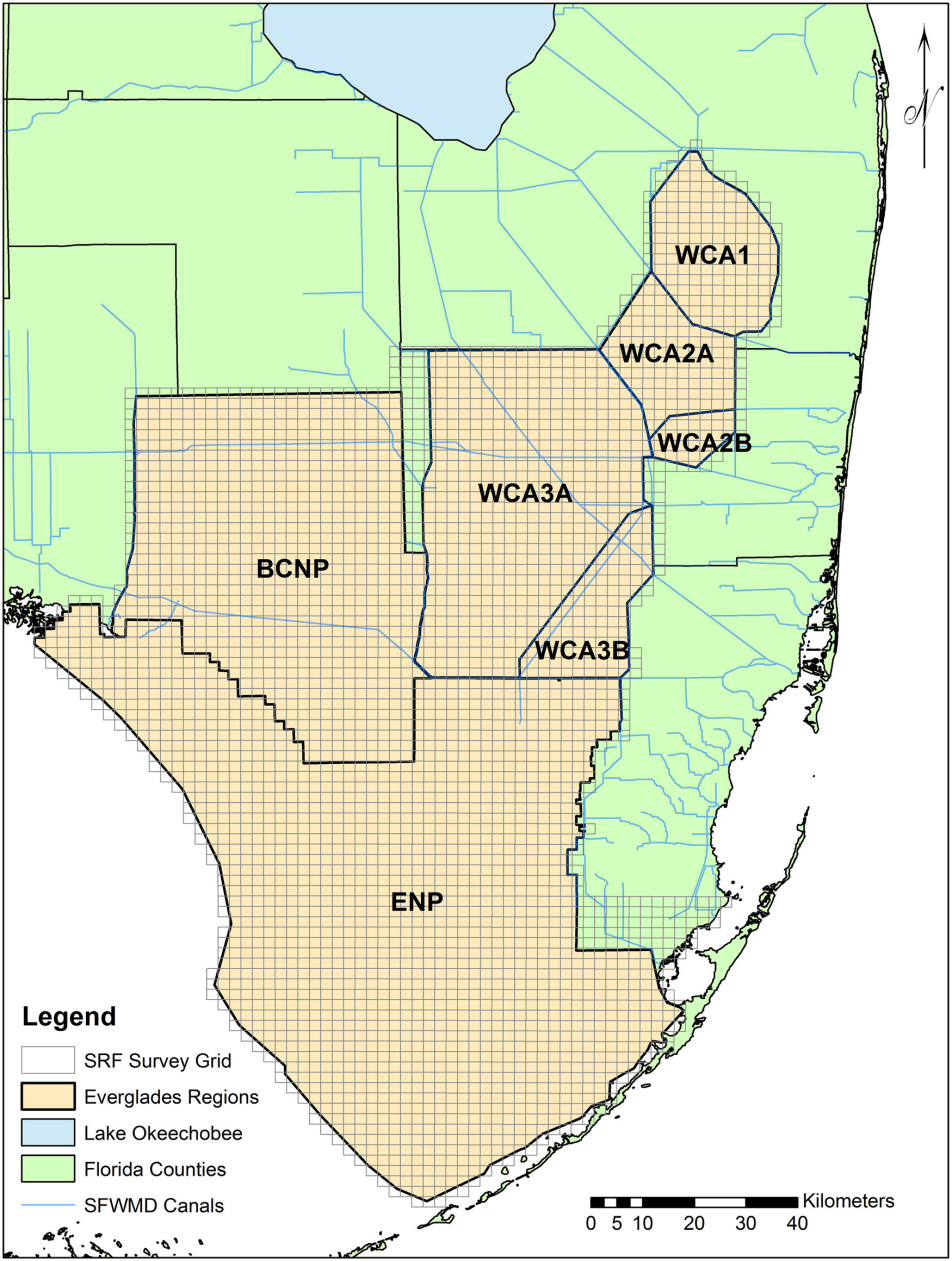
South Florida study system displaying Everglades hydrological basins (regions) and Systematic Reconnaissance Flight (SRF) survey extent. The regions of coverage include Water Conservation Areas (WCA) 1, 2A, 2B, 3A, 3B, Big Cypress National Park (BCNP), and Everglades National Park (ENP). South Florida Water Management District (SFWMD) canals are displayed for reference.

Egret, ibis, and stork foraging distributions and abundance were produced from Systematic Reconnaissance Flights (SRF; [28]) across the Greater Everglades system (Water Conservation Areas [WCA], Big Cypress National Park [BCNP] and Everglades National Park [ENP]; Fig 1). From January–June, during low altitude (61m) flights, two observers recorded the abundance, location, and species of wading birds detected in belt transects spaced at 2-km intervals [28]. This long-term and comprehensive dataset was ideal for quantifying habitat use by wading birds because it spans the extant Everglades and demonstrates the species-specific foraging response to hydrological variation over multiple years.

SRF data from 2000–2009 were overlaid on water depth data for the same dates that were derived from the Everglades Depth Estimation Network (EDEN; [29]). The EDEN is a nearly real-time integrated network of water gauges that calculates daily water depth (within ± 5 cm) in 400 m × 400 m grid cells accounting for evapotranspiration, rainfall, and sheet flow [30]. From EDEN, hydrological variables over multiple temporal scales and with existing links to wading bird responses were calculated as proxies for landscape processes that influence prey availability (hereafter “resources”). Days since drydown (DSD) quantified dynamics of long-term prey production by counting the number of consecutive days over 3 years that a cell had a water depth of greater than zero (i.e., length of inundation period). DSD is an important variable that accounts for prey biomass production in the wet season and availability in the dry season [31–32]. Furthermore, egrets and ibises selected for DSD in a year with good, but not poor, habitat conditions indicating it was likely a limiting resource [17]. The rate of receding water every two weeks during the dry season reflected the dynamics of hydrologic processes that concentrate prey. It was calculated by subtracting the water depth in a cell on a given day from the water depth 2 weeks prior and dividing by 14 days. The 2-week recession (i.e., drying) rate variable also predicts dry season biomass when incorporated with wet season biomass or days since drydown [32]. Described as the recession selectivity model, daily recession rate was selected by egrets and ibises in a year with poor habitat conditions, an adaptation to access new patches when prey was limiting [17]. Daily water depth quantified short-term availability of prey. Water depth is an important predictor of differential habitat selection in wading birds [13] and of relative reproductive success among species [24,26]. Using RSFs, the selection of water depths has been described for all species in this study [17,24]. We also calculated a dry to wet reversal variable to estimate the percentage of the availability window that had dried below the 10% quantile of depth use and rewetted for each species. This variable was necessary in predicting species distributions because cells could return to the suitable depth range after going dry, but would have severely depleted prey populations [33]. Hydroperiod was defined as the mean number of days per year (during the study period) that water depth of an EDEN cell was greater than zero. In addition to affecting fish density, hydroperiod affects wading bird distributions by influencing long-term changes in microtopography and vegetation communities [34].

Available habitat was quantified by identifying species-specific water depth ranges suitable for foraging. For egrets, ibises and storks, EDEN water depths at foraging observations were classified as suitable from the 10–90% quantiles of use (e.g., [35] to determine average resource levels considered available to foraging birds for the TFC model.

### Temporal Foraging Conditions (TFC; Patch Quality)

The SRF surveys from days in Jan–May, 2000–2009 (n = 243) were used to determine the daily abundance of foraging flocks and total individuals in the Everglades landscape. Flock presence was defined as one or more birds of the target species (egret, ibis and stork) detected in a given EDEN cell, whereas abundance of individual species considered the total number of birds present. For each species within available habitat of the daily SRF survey extent, we calculated the daily average of three temporally-specific EDEN hydrological variables (depth, recession rate, and days since drydown) as a proxy for prey dynamics. These resource levels and their spatial heterogeneity (SD) were employed to predict values of resource use by wading birds (i.e., selection), which were then used to predict daily flock and individual abundance.

Mean daily use values by wading birds were plotted as a function of mean daily availability of resources to determine the species’ selection response to a gradient of their environmental conditions. In addition, an *a priori* set of candidate models was tested in Proc Mixed [36] to determine if other hydrological variables were contributing to use of depth, recession and DSD. Models consisting of unique combinations of terms (available *Depth*, *Depth SD*, *Depth* ^*2*^; available *Recession*, *Recession SD*, *Recession* ^*2*^; available *DSD*, *DSD SD*, *DSD* ^*2*^), the interactions between the three resource terms, and interaction between depth availability and depth use (i.e., depth selection; for DSD use only) were evaluated for parsimony using Akaike’s Information Criterion for small sample sizes (AIC_c_; [37]). The random term *Month* was included to determine temporal effects of resource selection independent of hydrology. Models were retained if ΔAIC_c_ < 4 [37]. Model-averaged coefficients and standard errors (SE) were calculated for each fixed and random parameter by averaging all models containing the variable in proportion to the AIC weight. The importance of a specific term was determined by summing the weights of all models containing that term.

The daily abundance of flocks is correlated to the abundance of wading birds utilizing the landscape on any given day (mean R^2^ = 0.77). Both individual and flock responses were modeled because wading birds are highly social and select foraging habitat based in part on the presence of conspecifics, a process that may increase or decrease individual fitness [38]. Data for individuals and flocks across the landscape were all fourth-root transformed to meet assumptions of normality, with the exception of stork flocks, which were inverse fourth-root transformed because of the high occurrence of single flocks within SRF surveys. An *a priori* set of candidate models was tested in Proc Mixed [36] to determine how hydrological variables used by wading birds and the month of the dry season contributed to the flock and individual response across the landscape. Models consisting of unique combinations of terms (*Depth* use, *Depth* use^2^, available *Depth SD*; *Recession* use, *Recession* use^2^, available *Recession SD; DSD* use, *DSD* use^2^, available *DSD SD*; available dry to wet *Reversal*), the interactions between the three resources, and interaction between depth availability and depth use were evaluated for parsimony using Akaike’s Information Criterion for small sample sizes (AIC_c_; [37]). Models were evaluated similar to analyses mentioned above.

### Spatial Foraging Conditions (SFC; Patch Abundance)

Throughout the greater Everglades, SRF data from 2000–2009 were used to identify the number of instances over the period of record that a species used a given cell (i.e., frequency of use). Water depth, recession rate, DSD, dry to wet reversal, and hydroperiod use were then averaged over each instance of use, with the expectation that hydrological variables would converge on optimal values the more a cell was frequented.

Frequencies of cell use were all fourth-root transformed to meet assumptions of normality, with the exception of storks, which were inverse square-root transformed because of many single occurrences in a cell over the decade. An *a priori* set of candidate models was tested in Proc Glimmix [39] to determine how the aforementioned hydrological variables were contributing to frequency of locations used (i.e., cells). Models consisting of unique combinations of terms (*Depth*, *Depth* ^*2*^, *Recession*, *Recession* ^*2*^, *DSD*, *DSD* ^*2*^, *Reversal*, *Hydroperiod (HP)* and *HP* ^2^); the interactions among depth, recession, DSD; and the random effect *Region* (WCA, BCNP, and ENP; see Fig 1) were evaluated for parsimony using Akaike’s Information Criterion for small sample sizes (AIC_c_; [36]).

A challenge in interpreting the results of spatial SDMs can occur when spatial autocorrelation is prevalent in resources. Spatial models that incorporate autocorrelation are more likely to avoid consequences of pseudoreplication and represent biological processes unaccounted for by the environmental covariates [40]. Proc Glimmix was utilized specifically because it can account for residual spatial correlation using a non-parametric radial smoother. It uses a random effect to model complicated trend surfaces (such as a spatial grid) without having to specify a particular parametric functional form. The net effect of including a random effect for this spatial correlation is a semi-parametric model where the residuals in a landscape are mostly free of spatial correlation [41]. By capturing patterns in the spatial variation of the landscape through radial smoothing, the noise independent of the hydrologic parameters can be reduced to better capture the species-specific behavioral response to rapidly changing habitat conditions [40]. Semi-parametric models were retained if ΔAIC_c_ < 4 [36]. Model-averaged coefficients and SE were calculated for each fixed and random parameter by averaging all models containing the variable in proportion to the AIC weight. The importance of a specific term was determined by summing the weights of all models containing that term.

## Results

### Hydrological Variables and Habitat Use

Egrets used a mean water depth of 19.68 (±0.07 SE) cm and habitat availability (10% to 90% quantile) was defined by the range -1.78–41.63 cm. This is the largest range of depths of the three species and supports the egret’s tolerance of a wider range of water depths [13,17]. Ibises used a mean water depth of 13.40 cm (±0.09 SE) and available habitat was restricted to a depth range of -4.95–32.62 cm (10%– 90% quantile). Storks used a mean water depth of 12.86 (±0.29 SE) and available habitat was defined by the depth range -8.73–35.77 cm (10%– 90% quantile).

The model that best explained egret depth use included the terms for Depth, Depth SD, Depth^2^ and Recession (Table 1). Egrets used shallower locations in a dryer landscape when less heterogeneity occurred within their depth range. Also, egrets used shallower locations with increasing recession rates. The top model accounted for 81% of the variation in depth use with all variables exhibiting high importance (>70%; Table 1). The most parsimonious model describing depth use by ibis included the terms for Depth, Depth SD, and the Recession*DSD interaction (Table 1). Similar to the egret, ibis used shallower locations as the landscape dried and variability decreased. The interaction term indicated a greater selection for shallow water as recession rates increased when DSD availability was high, as ibis targeted sites with highly concentrated prey. Ibis used deeper water in January and February and shallower water in April and May. The top model explained 79% of the variation in depth use with all of the terms exhibiting high importance (>70%; Table 1). Stork depth use was best explained by Depth, Depth^2^, DSD SD, and the Depth*DSD interaction, with selection for shallower locations when DSD availability and heterogeneity were high. The top model accounted for 78% of the variation in depth use and all terms were of high importance (Table 1).

**Table 1:** Ranking of candidate models describing variables influencing daily mean depth use of Great Egrets, White Ibises, and Wood Storks in the Florida Everglades (Proc Mixed).

**GREAT EGRET MODEL**	**K^b^**	**AIC_c_^c^**	**modelid**	**ΔAIC_c_^d^**	**weight**	**R^2^**
**Depth, Depth SD, Depth^2^, Recess**	7	1588.7	**15**	**0.00**	**0.22**	**0.81**
Depth, Depth SD, Depth^2^, Recess, DSD, DSD^2^	9	1589.0	20	0.23	0.19	
Depth, Depth^2^, Recess, Depth*Recess	7	1589.9	23	1.15	0.12	
**Variable**	**N**	**Avg PE**	**SE**	**Importance**		
**Intercept**	**27**	**-18.875**	**2.83**	**1.00**		
**Depth**	**24**	**2.300**	**0.42**	**1.00**		
**Recess**	**16**	**-1.652**	**1.16**	**0.94**		
**Depth SD**	**14**	**0.614**	**0.33**	**0.91**		
**Depth^2^**	**10**	**-0.017**	**0.01**	**0.84**		
**WHITE IBIS MODEL**	**K^b^**	**AIC_c_^c^**	**modelid**	**ΔAIC_c_^d^**	**weight**	**R^2^**
Depth, Depth SD, Recess*DSD	**6**	**1472.6**	**3**	**0.00**	**0.30**	**0.79**
Depth, Depth SD, Recess, Recess*DSD	7	1473.3	21	0.71	0.21	
Depth, Depth^2^, Recess, Recess*DSD	7	1475.0	14	2.43	0.09	
Depth, Depth SD, Recess, Recess*DSD, Cells	8	1475.3	26	2.78	0.07	
**Variable**	**N**	**Avg PE**	**SE**	**Importance**		
**Intercept**	**27**	**-12.304**	**2.61**	**1.00**		
**Depth**	**24**	**1.726**	**0.21**	**1.00**		
Recess*DSD	**14**	**-0.004**	**0.00**	**0.87**		
**Depth SD**	**12**	**0.558**	**0.24**	**0.84**		
**WOOD STORK MODEL**	**K^b^**	**AIC_c_^c^**	**modelid**	**ΔAIC_c_^d^**	**weight**	**R^2^**
Depth, Depth^2^, DSD SD, Depth*DSD	**6**	**1229.2**	**26**	**0.00**	**0.18**	**0.78**
Depth, Depth^2^, Recess, DSD SD, Depth*DSD	7	1229.6	21	0.43	0.15	
Depth, Depth^2^, Recess^2^, DSD SD, Depth*DSD	7	1229.8	22	0.67	0.13	
Depth, Depth^2^, DSD SD, Depth*DSD, Recess*DSD	7	1230.3	16	1.16	0.10	
**Variable**	**N**	**Avg PE**	**SE**	**Importance**		
**Intercept**	**27**	**-19.170**	**3.24**	**1.00**		
**Depth**	**22**	**3.199**	**0.28**	**1.00**		
**Depth^2^**	**22**	**-0.037**	**0.01**	**1.00**		
Depth*DSD	**14**	**-0.001**	**0.00**	**0.95**		
**DSD SD**	**14**	**0.012**	**0.01**	**0.80**		

Models are ranked by differences in Akaike’s information criterion and only candidate models within ΔAIC_c_^d^ ≤ 4.0 are presented. Model selection results are followed by model averaging results for each species. The R^2^ represents the model fit for the estimated mean daily depth use vs. model averaged predicted values.

The model that best explained egret recession rate use included the terms for Recession, Recession SD, Depth SD, and the Depth*Recession interaction (Table 2). Egret recession rate use increased with increasing recession rate availability and heterogeneity, and a decrease in depth heterogeneity (as the landscape dries). Higher recession rates were selected for in shallow water. This model explained 95% of the variation in recession rate use. The most parsimonious model describing recession rate use by ibis included the terms Recession, Recession SD, and the Depth*Recession interaction (Table 2). A competing model with similar weight also included Recession^2^; however, the parameter estimate plus the SE overlaps zero suggesting a weak effect. Ibis recession rate use also increased with increasing recession rate availability and heterogeneity with a selection for higher rates in a dryer landscape. The top model accounted for 95% of the variation in recession rate use. Recession rate use by storks was best explained by the terms Recession, Recession SD, Depth SD, and Depth*Recession. The model explained 85% of the variation in recession rate use (Table 2). Recession rate use increased with increasing recession rate availability and heterogeneity and decreasing depth heterogeneity. Similar to the other species, selection for more rapid recession rate occurred when landscape depth was low. No variation was evident in recession rate use across months, so the term was removed.

**Table 2:** Ranking of candidate models describing variables influencing daily mean 2-week recession rate use of Great Egrets, White Ibises, and Wood Storks in the Florida Everglades (Proc Mixed).

**GREAT EGRET MODEL**	**K^b^**	**AIC_c_^c^**	**modelid**	**ΔAIC_c_^d^**	**w_i_^e^**	**R^2^**
Recess, Recess SD, Depth SD, Depth*Recess	**6**	**-337.7**	**23**	**0.00**	**0.44**	**0.95**
Recess, Recess SD, Recess^2^, Depth SD, Depth*Recess, Recess*DSD	8	-335.5	9	2.23	0.14	
Recess, Recess SD, Recess, Depth, Depth SD, Depth*Recess, Cells	9	-334.9	8	2.85	0.11	
**Variable**	**N**	**Avg PE**	**SE**	**Importance**		
**Intercept**	**27**	**0.123**	**0.04**	**1.00**		
**Recess**	**16**	**1.074**	**0.04**	**1.00**		
**Recess SD**	**10**	**0.247**	**0.05**	**1.00**		
**Depth SD**	**10**	**-0.012**	**0.00**	**0.98**		
Depth*Recess	**10**	**-0.004**	**0.00**	**0.83**		
**WHITE IBIS MODEL**	**K^b^**	**AIC_c_^c^**	**modelid**	**ΔAIC_c_^d^**	**w_i_^e^**	**R^2^**
Recess, Recess SD, Depth*Recess	**5**	**-281.5**	**23**	**0.00**	**0.34**	**0.95**
Recess, Recess SD, Recess^2^, Depth*Recess	6	-281.1	8	0.46	0.27	
Recess, Recess SD, Recess^2^, Depth SD, DSD, Depth*DSD, Depth*Recess, Depth*DSD	9	-279.5	26	2.01	0.13	
**Variable**	**N**	**Avg PE**	**SE**	**Importance**		
**Intercept**	**27**	**0.017**	**0.02**	**1.00**		
**Recess**	**16**	**1.156**	**0.04**	**1.00**		
**Recess SD**	**10**	**0.150**	**0.11**	**1.00**		
Depth*Recess	**10**	**-0.013**	**0.05**	**1.00**		
**WOOD STORK MODEL**	**K^b^**	**AIC_c_^c^**	**modelid**	**ΔAIC_c_^d^**	**w_i_^e^**	**R^2^**
Recess, Recess SD, Depth SD, Depth*Recess	**6**	**-46.9**	**24**	**0.00**	**0.33**	**0.85**
Recess, Recess SD, Recess^2^, Depth SD, Depth*Recess, Recess*DSD	8	-46.5	26	0.41	0.27	
Recess, Recess SD, Depth SD, Depth*Recess, Recess*DSD	7	-45.0	9	1.83	0.13	
Recess, Recess SD, Depth*Recess	5	-45.0	23	1.91	0.13	
**Variable**	**N**	**Avg PE**	**SE**	**Importance**		
**Intercept**	**27**	**0.055**	**0.08**	**1.00**		
**Recess**	**18**	**1.128**	**0.07**	**1.00**		
**Recess SD**	**12**	**0.560**	**0.11**	**1.00**		
Depth*Recess	**10**	**-0.010**	**0.00**	**1.00**		
**Depth SD**	**12**	**-0.011**	**0.00**	**0.97**		

Models are ranked by differences in Akaike’s information criterion and only candidate models within ΔAIC_c_^d^ ≤ 4.0 are presented. Model selection results are followed by model averaging results for each species. The R^2^ represents the model fit for the estimated mean daily recession rate use vs. model averaged predicted values.

The model that best explained DSD use by egrets included the terms for Depth, Depth SD, Depth^2^, DSD, DSD SD, Depth*Depth Use, Depth*DSD and Recession*DSD (Table 3). Accounting for DSD availability, DSD selection increased with increasing landscape depths and lower depth heterogeneity. Longer DSD use was evident when egrets were using deeper water, but primarily in mean available depths > 15 cm when DSD selection was high. DSD selection was also higher as recession rates slowed and DSD use increased with higher DSD heterogeneity. The top model explained 92% of the variation in DSD use. The most parsimonious model describing ibis DSD use included the terms for Depth, Depth^2^, DSD, DSD SD, Depth*Depth Use, and Depth*DSD (Table 3). Accounting for DSD availability, use of longer DSD occurred in a wetter landscape, an effect strengthened when ibis used deeper water. Similar to egrets, in mean available depths > 10 cm, there was greater selection for long DSD cells. DSD use also increased as available DSD and heterogeneity increased. The top model accounted for 92% of the variation in DSD use. DSD use by storks was best explained by the terms Depth, Depth^2^, DSD, DSD SD and Depth Use*Depth (Table 3). Storks used longer DSD cells when DSD availability and heterogeneity were high and in a wetter landscape when feeding in deeper water. No variation was evident in DSD use across months, so the term was removed.

**Table 3:** Ranking of candidate models describing variables influencing daily mean days since drydown (DSD) use of Great Egrets, White Ibises, and Wood Storks in the Florida Everglades (Proc Mixed).

**GREAT EGRET MODEL**	**K^b^**	**AIC_c_^c^**	**modelid**	**ΔAIC_c_^d^**	**w_i_^e^**	**R^2^**
Depth, Depth SD, Depth^2^, DSD, DSD SD, Depth Use*Depth, Depth*DSD, Recess*DSD	**10**	**2676.7**	**9**	**0.00**	**0.88**	**0.92**
Depth, Depth SD, Depth^2^, DSD, DSD SD, DSD, ^2^Depth Use*Depth, Depth*DSD, Depth*Recess, Recess*DSD	12	2680.7	12	4.08	0.11	
**Variable**	**N**	**Avg PE**	**SE**	**Importance**		
**Intercept**	**27**	**-134.269**	**25.47**	**1.00**		
**Depth**	**16**	**33.356**	**3.57**	**1.00**		
**Depth SD**	**10**	**-6.146**	**2.98**	**1.00**		
**Depth^2^**	**10**	**-1.687**	**0.12**	**1.00**		
**DSD**	**14**	**0.483**	**0.08**	**1.00**		
**DSD SD**	**12**	**0.171**	**0.07**	**1.00**		
Depth Use* Depth	**10**	**0.554**	**0.04**	**1.00**		
Depth*DSD	**14**	**0.021**	**0.00**	**1.00**		
Recess*DSD	**14**	**-0.069**	**0.04**	**1.00**		
**WHITE IBIS MODEL**	**K^b^**	**AIC_c_^c^**	**modelid**	**ΔAIC_c_^d^**	**w_i_^e^**	**R^2^**
Depth, Depth^2^, DSD, DSD SD, Depth Use*Depth, Depth*DSD	**8**	**2609.1**	**9**	**0.00**	**0.51**	**0.92**
Depth, Depth^2^, DSD, DSD SD, Depth Use*Depth, Depth*DSD, Cells	9	2609.6	15	0.51	0.40	
**Variable**	**N**	**Avg PE**	**SE**	**Importance**		
**Intercept**	**27**	**-96.078**	**23.19**	**1.00**		
**Depth**	**18**	**26.009**	**2.89**	**1.00**		
**Depth^2^**	**10**	**-1.802**	**0.15**	**1.00**		
**DSD**	**14**	**0.548**	**0.08**	**1.00**		
**DSD SD**	**10**	**0.122**	**0.06**	**1.00**		
Depth Use*Depth	**12**	**0.638**	**0.05**	**1.00**		
Depth*DSD	**14**	**0.024**	**0.00**	**1.00**		
**WOOD STORK MODEL**	**K^b^**	**AIC_c_^c^**	**modelid**	**ΔAIC_c_^d^**	**w_i_^e^**	**R^2^**
Depth, Depth^2^, DSD, DSD SD, Depth Use*Depth	**7**	**2149.0**	**19**	**0.00**	**0.43**	**0.76**
Depth, Depth^2^, DSD, DSD SD, Depth Use*Depth, Depth*Recess	8	2150.5	9	1.45	0.21	
Depth, Depth^2^, DSD, DSD SD, Depth Use*Depth, Depth*DSD	8	2150.7	15	1.64	0.19	
**Variable**	**N**	**Avg PE**	**SE**	**Importance**		
**Intercept**	**27**	**-79.957**	**38.01**	**1.00**		
**DSD**	**14**	**0.809**	**0.08**	**1.00**		
**Depth**	**18**	**17.188**	**3.47**	**1.00**		
**DSD SD**	**10**	**0.246**	**0.11**	**1.00**		
Depth Use*Depth	**12**	**0.418**	**0.06**	**1.00**		
**Depth^2^**	**10**	**-0.930**	**0.13**	**1.00**		

Models are ranked by differences in Akaike’s information criterion and only candidate models within ΔAIC_c_^d^ ≤ 4.0 are presented. Model selection results are followed by model averaging results for each species. The R^2^ represents the model fit for the estimated mean daily DSD use vs. model averaged predicted values.

### Temporal Foraging Conditions (Patch Quality)

**Fig 2:**
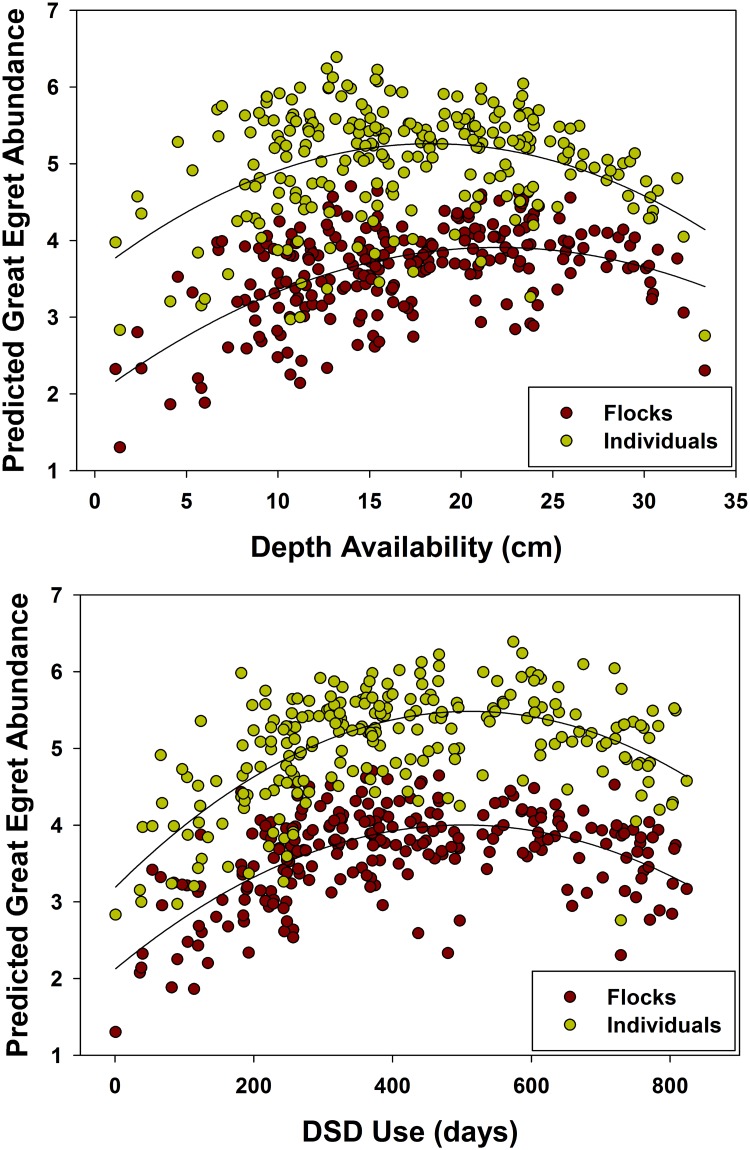
Daily mean landscape flocks and individuals (fourth-root transformed) predicted by the model-averaged terms for the Great Egret. Predicted presence and abundance are highest with an average landscape depth (within available depths) of 15–22 cm and when Great Egrets are using an average DSD of ~500 days.

Flocks of egrets across the landscape are best explained by Depth SD, Depth^2^, DSD SD, DSD^2^, Reversal, and Depth Use*Depth. The second best model included the term for Depth*DSD, but this was a term with low importance (Table 4). The top model for egret abundance included the terms Depth availability, Depth SD, Depth^2^, DSD SD, Reversal, and Depth Use*Depth (Table 5). Egret flock and individual abundance increased with increasing DSD use (Fig 2) and heterogeneity, in a similar form to the response of small fish density to days since last drydown [42]. The depth terms indicated higher flock and individual abundance when depth heterogeneity was high and when egrets were selecting deeper water. Furthermore, individual abundance increased as the landscape dried, whereas this term was not present in the flocks model (Fig 2). Flocks and individuals were negatively impacted by dry to wet reversals. Ibis landscape flocks were best explained by Depth SD, DSD, DSD SD, DSD^2^, and Depth*DSD (Table 4). A competing model with less support included the term Depth; however, with low variable importance, this term was not considered. The top model for individual abundance included the terms Depth availability, Depth SD, Depth^2^, Recession SD, DSD, DSD^2^, Reversal, and Depth Use*Depth (Table 5).

**Table 4:** Ranking of candidate models describing variables influencing daily mean flock abundance of Great Egrets, White Ibises, and Wood Storks in the Florida Everglades (Proc Mixed).

**GREAT EGRET MODEL**	**K^b^**	**AIC_c_^c^**	**modelid**	**ΔAIC_c_^d^**	**w_i_^e^**	**R^2^**
Depth SD, Depth^2^, DSD SD, DSD^2^, Reversal, Depth Use*Depth	**8**	**626.3**	**5**	**0.00**	**0.41**	**0.39**
Depth SD, Depth^2^, DSD SD, Reversal, Depth*DSD, Depth Use*Depth	8	628.45	9	0.16	0.38	
**Variable**	**N**	**Avg PE**	**SE**	**Importance**		
**Intercept**	**27**	**1.132**	**0.33**	**1.00**		
**Depth SD**	**10**	**0.194**	**0.03**	**1.00**		
**Depth^2^**	**10**	**-0.002**	**0.00**	**1.00**		
**DSD SD**	**10**	**0.004**	**0.00**	**1.00**		
**Reversal**	**14**	**-1.675**	**0.30**	**1.00**		
Depth Use*Depth	**18**	**0.003**	**0.00**	**1.00**		
**DSD^2^**	**16**	**-0.000**	**0.00**	**0.62**		
**WHITE IBIS MODEL**	**K^b^**	**AIC_c_^c^**	**modelid**	**ΔAIC_c_^d^**	**w_i_^e^**	**R^2^**
Depth SD, DSD, DSD SD, DSD^2^, Depth*DSD	**7**	**558.6**	**15**	**0.00**	**0.70**	**0.32**
Depth, Depth SD, DSD, DSD SD, DSD^2^, Depth*DSD	8	560.7	17	2.01	0.26	
**Variable**	**N**	**Avg PE**	**SE**	**Importance**		
**Intercept**	**27**	**0.453**	**0.32**	**1.00**		
**Depth SD**	**10**	**0.128**	**0.04**	**1.00**		
**DSD**	**10**	**0.005**	**0.00**	**1.00**		
**DSD^2^**	**10**	**0.000**	**0.00**	**1.00**		
**DSD SD**	12	0.003	0.00	1.00		
Depth*DSD	14	-0.000	0.00	0.95		
**WOOD STORK MODEL**	**K^b^**	**AIC_c_^c^**	**modelid**	**ΔAIC_c_^d^**	**w_i_^e^**	**R^2^**
**Depth A, Depth SD, Depth^2^, Recess^2^, Reversal**, Depth Use*Depth	**8**	**-211.8**	**21**	**0.00**	**0.77**	**0.44**
Depth A, Depth, Depth SD, Depth^2^, Recess^2^, Reversal, DSD^2^, Depth Use*Depth	10	-209.4	14	2.45	0.23	
**Variable**	**N**	**Avg PE**	**SE**	**Importance**		
**Intercept**	**27**	**0.578**	**0.05**	**1.00**		
**Depth A**	**10**	**0.024**	**0.00**	**1.00**		
**Depth SD**	**12**	**-0.032**	**0.01**	**1.00**		
**Depth^2^**	**10**	**0.000**	**0.00**	**1.00**		
**Recess^2^**	**14**	**0.053**	**0.02**	**1.00**		
**Reversal**	**10**	**0.127**	**0.08**	**1.00**		
Depth Use*Depth	**12**	**-0.001**	**0.00**	**1.00**		

Models are ranked by differences in Akaike’s information criterion and only candidate models within ΔAIC_c_^d^ ≤ 4.0 are presented. Model selection results are followed by model averaging results for each species. The R^2^ represents the model fit for the estimated daily flock abundance vs. model averaged predicted values.

**Table 5:** Ranking of candidate models describing variables influencing daily individual abundance of Great Egrets, White Ibises, and Wood Storks in the Florida Everglades (Proc Mixed).

**GREAT EGRET MODEL**	**K^b^**	**AIC_c_^c^**	**modelid**	**ΔAIC_c_^d^**	**w_i_^e^**	**R^2^**
Depth A, Depth SD, Depth^2^, DSD SD, Reversal, Depth Use*Depth	**9**	**797.6**	**12**	**0.00**	**0.44**	**0.35**
Depth A, Depth SD, Depth^2^, Recess^2^, DSD SD, Reversal, Depth Use*Depth	9	797.6	2	0.08	0.42	
**Variable**	**N**	**Avg PE**	**SE**	**Importance**		
**Intercept**	**27**	**3.176**	**0.54**	**1.00**		
**Reversal**	**10**	**-2.482**	**0.44**	**1.00**		
Depth Use*Depth	**11**	**0.005**	**0.00**	**1.00**		
**Depth SD**	**9**	**0.268**	**0.05**	**1.00**		
**DSD SD**	**9**	**0.005**	**0.00**	**1.00**		
**Depth^2^**	**11**	**-0.003**	**0.00**	**1.00**		
**Depth A**	**9**	**-0.109**	**0.04**	**1.00**		
**WHITE IBIS MODEL**	**K^b^**	**AIC_c_^c^**	**modelid**	**ΔAIC_c_^d^**	**w_i_^e^**	**R^2^**
Depth A, Depth SD, Depth^2^, Recess SD, DSD, DSD^2^, Reversal, Depth Use*Depth	**10**	**888.3**	**4**	**0.00**	**0.75**	**0.31**
Depth SD, Depth^2^, Recess SD, DSD, Reversal, DSD Use*DSD	8	891.3	11	2.98	0.17	
**Variable**	**N**	**Avg PE**	**SE**	**Importance**		
**Intercept**	**27**	**2.764**	**0.72**	**1.00**		
**DSD**	**13**	**0.008**	**0.00**	**1.00**		
**Reversal**	**9**	**-2.946**	**0.66**	**1.00**		
**Depth SD**	**9**	**0.336**	**0.10**	**1.00**		
**Recess SD**	11	1.590	0.63	1.00		
**Depth^2^**	9	-0.003	0.00	1.00		
**Depth A**	9	-0.167	0.08	0.87		
**DSD^2^**	10	0.000	0.00	0.76		
Depth Use*Depth	9	0.007	0.00	0.76		
**WOOD STORK MODEL**	**K^b^**	**AIC_c_^c^**	**modelid**	**ΔAIC_c_^d^**	**w_i_^e^**	**R^2^**
Depth A, Depth SD, Depth^2^, Recess SD, Recess^2^, Reversal, Depth Use*Depth	**9**	**481.1**	**5**	**0.00**	**0.79**	**0.30**
Depth A, Depth, Depth SD, Depth^2^, Recess, Recess SD, Recess^2^, DSD SD, Reversal, Depth Use*Depth	12	484.9	6	3.72	0.12	
**Variable**	**N**	**Avg PE**	**SE**	**Importance**		
**Intercept**	**27**	**2.794**	**0.39**	**1.00**		
**Depth SD**	**11**	**0.141**	**0.38**	**1.00**		
**Depth A**	**10**	**-0.114**	**0.03**	**1.00**		
**Reversal**	**9**	**-1.471**	**0.58**	**0.99**		
**Recess SD**	**9**	**0.907**	**0.48**	**0.98**		
**Recess^2^**	**13**	**-0.338**	**0.13**	**0.98**		
Depth Use*Depth	**9**	**0.003**	**0.00**	**0.94**		
**Depth^2^**	**9**	**-0.001**	**0.00**	**0.94**		

Models are ranked by differences in Akaike’s information criterion and only candidate models within ΔAIC_c_^d^ ≤ 4.0 are presented. Model selection results are followed by model averaging results for each species. The R^2^ represents the model fit for the estimated daily individual abundance vs. model averaged predicted values.

**Fig 3:**
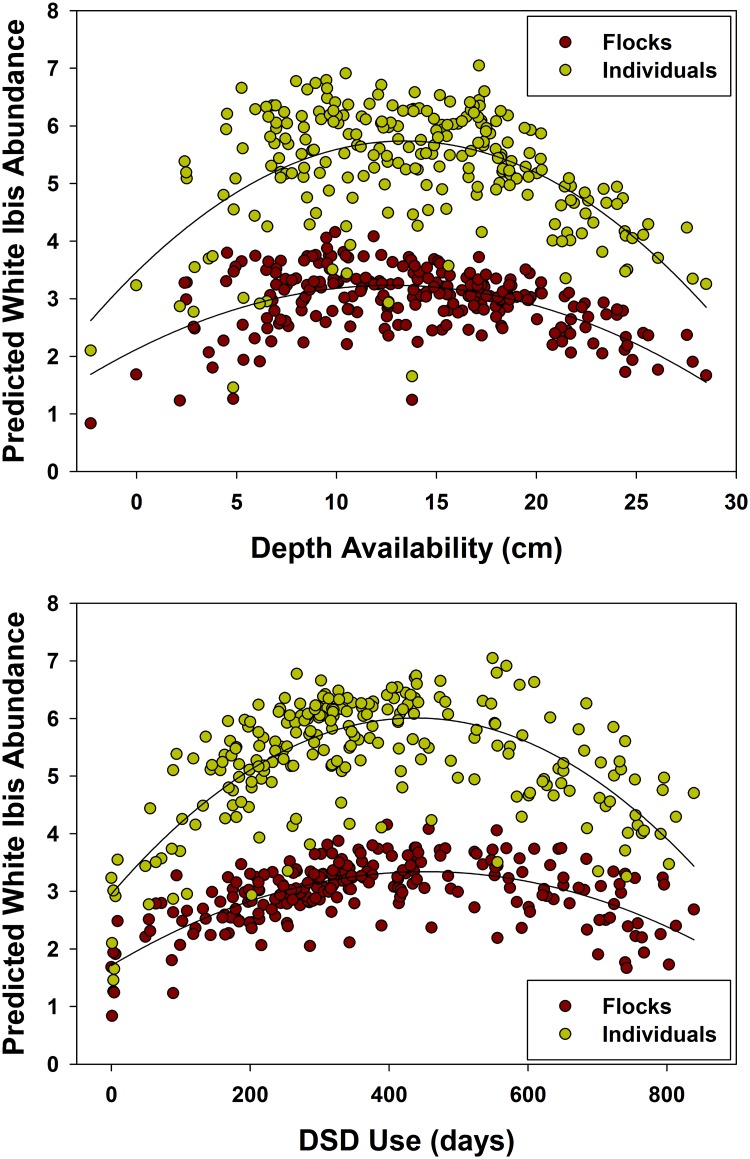
Daily mean landscape flocks and individuals (fourth-root transformed) predicted by the model-averaged terms for the White Ibis. Predicted presence and abundance are highest with an average landscape depth (within available depths) of 10–17 cm and when White Ibis are using an average DSD of ~450 days.

Similar to the egret, ibis flock and individual abundance increased with increasing DSD use, but started to decline more rapidly with DSD above 450 days (Fig 3), and this was pronounced in shallow water. Also, flocks and individuals increased as depth heterogeneity increased, occurring at intermediate landscape depths (Fig 3). Individual abundance declined with increasing reversals, whereas this variable had no effect on flock abundance.

**Fig 4:**
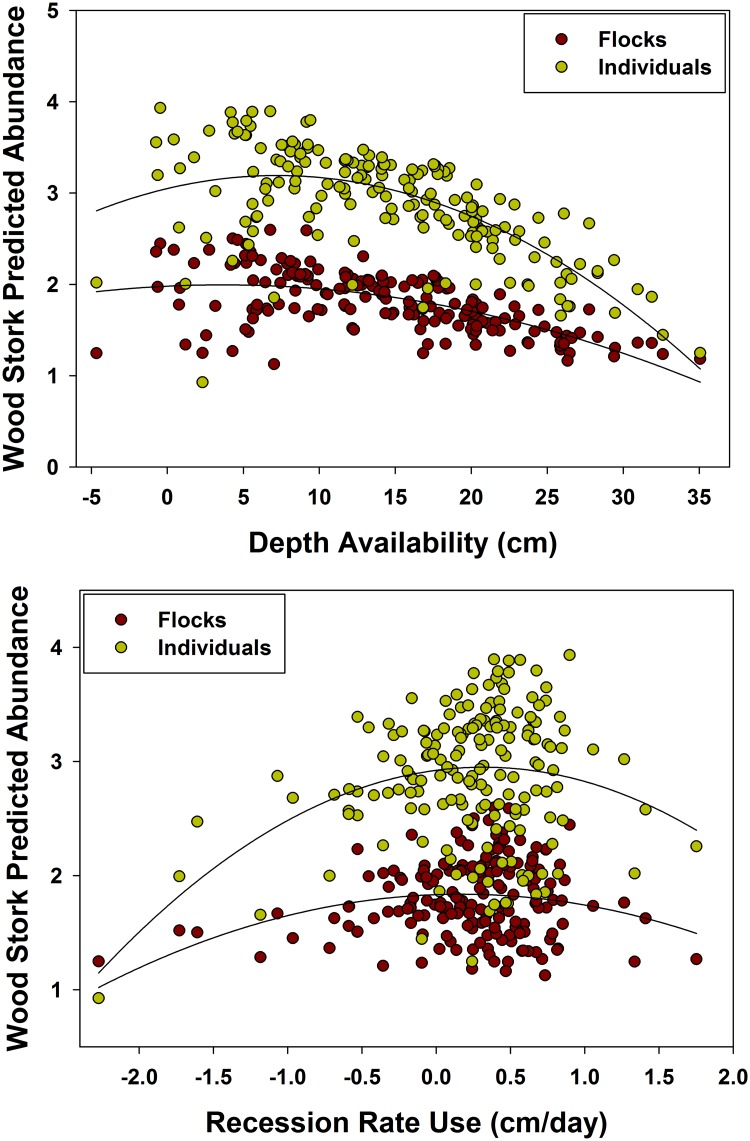
Daily mean landscape flocks and individuals (fourth-root transformed) predicted by the model-averaged terms for the Wood Stork. Predicted presence and abundance are highest with an average landscape depth (within available depths) less than 15 cm and when Wood Storks are using an average recession rate of ~0.5 cm/day.

Stork flocks across the landscape were best explained by Depth availability, Depth SD, Depth^2^, Recession^2^, Reversal, and Depth use*Depth (Table 4). The best model for stork individuals included the terms Depth availability, Depth SD, Depth^2^, Recession SD, Recession^2^, Reversal, and Depth use*Depth (Table 5). Predicted flock and individual abundance of storks increased with a drying landscape (Fig 4) and increasing depth heterogeneity, which was highest at intermediate landscape depths (peaking at ~15 cm). Similar to the egret, stork flocks and individuals increased when they were selecting deeper water. Flock and individual abundance also increased at intermediate recession rate use (peaking at ~0.5 cm/day; Fig 4) and with fewer reversals.

### Spatial Foraging Conditions (Patch Abundance)

**Fig 5:**
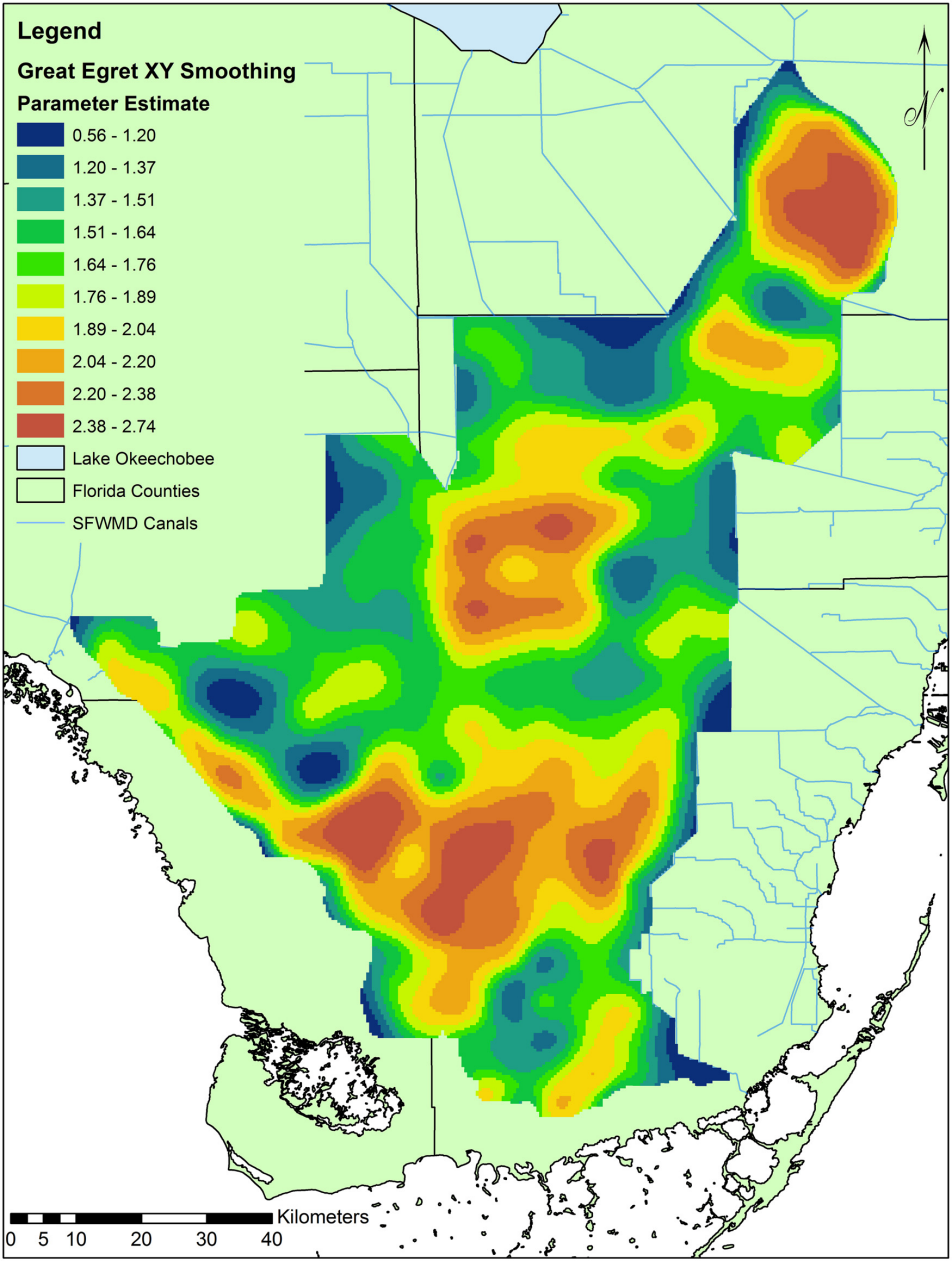
Map displaying XY parameter estimates accounting for residual spatial correlation of Great Egret frequency of use. Locations in red are frequented more often across time after accounting for hydrological predictors. South Florida Water Management District (SFWMD) canals are displayed for reference.

The model that best explained the frequency of cell use (i.e., spatial occurrence) by egrets contained the terms Depth, Depth^2^, DSD, DSD^2^, HP, Depth*DSD, and Recession*DSD (Table 6). The quadratic depth terms indicated that frequency of use was highest at intermediate values. Use of cells increased with increasing hydroperiod and peaked at an intermediate DSD, again suggesting an optimal DSD of ~500 days. Cell use increased with decreasing depth only when DSD use was high, as shallow water is only beneficial to egrets when prey is highly concentrated. Use was also more positively influenced by higher recession rate use in short versus long DSD cells. Taken together, these results suggest that the frequency of cell use increased more rapidly with increasing recession in cells with fewer DSD, yet these cells were still used at a lower mean frequency than cells where egrets were using greater depths and long DSD. Because recession rate use was strongly related to landscape recession rates (Table 2), rapid landscape recession rates likely increased cell use (and likely prey density) when DSD use was low to a level similar to that when DSD was high. After accounting for residual spatial correlation (see Fig 5), the model suggests that egrets more frequently used cells in the ENP region and slightly less frequently in the BCNP region.

**Table 6:** Ranking of candidate models describing variables influencing frequency of cell use (i.e., spatial occurrence) over the study period for the Great Egret, White Ibis, and Wood Stork (Proc Glimmix).

**GREAT EGRET MODEL**	**N**	**AICC**	**modelid**	**d_aic**	**weight**	**R^2^**
Depth, Depth^2^, DSD, DSD^2^, HP, Depth*DSD, Recess*DSD	**12**	**880.59**	**5**	**0.00**	**0.74**	**0.88**
Depth, Depth^2^, DSD, DSD^2^, Reversal, HP, Depth*DSD, Depth*Recess, Recess*DSD	14	883.83	18	3.24	0.15	
**Variable**	**N**	**Avg PE**	**SE**	**Importance**		
**Intercept**	**27**	**-0.584**	**7.48**	**1.00**		
**Depth**	**14**	**-0.006**	**0.00**	**1.00**		
**Depth^2^**	**14**	**-0.000**	**0.00**	**1.00**		
Depth*DSD	**15**	**0.000**	**0.00**	**1.00**		
**DSD^2^**	**15**	**-0.000**	**0.00**	**1.00**		
**HP**	**14**	**0.004**	**0.00**	**1.00**		
**DSD**	**13**	**0.001**	**0.00**	**0.99**		
Recess*DSD	**11**	**-0.000**	**0.00**	**0.99**		
**WHITE IBIS MODEL**	**N**	**AICC**	**modelid**	**d_aic**	**weight**	**R^2^**
Depth^2^, Recess, Recess^2^, Depth*DSD, Depth*Recess	**8**	**750.2**	**4**	**0.00**	**0.55**	**0.83**
Depth^2^, Recess, Recess^2^, DSD^2^, Depth*DSD	8	750.9	6	0.67	0.39	
**Variable**	**N**	**Avg PE**	**SE**	**Importance**		
**Intercept**	**27**	**1.126**	**7.79**	**1.00**		
**Depth^2^**	**17**	**-0.000**	**0.00**	**1.00**		
**Recess^2^**	**15**	**-0.012**	**0.01**	**1.00**		
**Recess**	**12**	**0.061**	**0.02**	**0.99**		
Depth*DSD	**16**	**0.000**	**0.00**	**0.94**		
Depth*Recess	**15**	**0.000**	**0.00**	**0.58**		
**WOOD STORK MODEL**	**N**	**AICC**	**modelid**	**d_aic**	**weight**	**R^2^**
Depth, Depth^2^, Recess^2^, DSD, DSD^2^, HP, HP^2^, Depth*DSD	**13**	**-485.9**	**12**	**0.00**	**0.49**	**0.55**
Depth, Depth^2^, Recess^2^, DSD, DSD^2^, Reversal, HP, HP^2^, Depth*DSD	14	-485.1	18	0.72	0.34	
**Variable**	**N**	**Avg PE**	**SE**	**Importance**		
**Intercept**	**27**	**0.987**	**0.63**	**1.00**		
**Depth**	**15**	**0.004**	**0.00**	**1.00**		
**Depth^2^**	**15**	**0.000**	**0.00**	**1.00**		
**DSD**	**13**	**-0.001**	**0.00**	**1.00**		
**DSD^2^**	**14**	**0.000**	**0.00**	**1.00**		
Depth*DSD	**13**	**-0.000**	**0.00**	**1.00**		
**HP^2^**	**9**	**-0.000**	**0.00**	**1.00**		
**HP**	**15**	**0.001**	**0.00**	**1.00**		

Models are ranked by differences in Akaike’s information criterion and only candidate models within ΔAIC_c_^d^ ≤ 4.0 are presented. Model selection results are followed by model averaging results for each species. The R^2^ represents the model fit for the estimated spatial occurrence vs. model averaged predicted values.

The most parsimonious ibis model explaining frequency of use included the terms Depth^2^, Recession, Recession^2^, Depth*DSD, and Depth*Recession (Table 6). The quadratic depth term shows that cell use frequency was highest in water shallower than the optimum for egrets, but ibis frequency still declined slightly if cells were too dry. The squared recession term again indicated high use at intermediate recession rates (~0.5 cm/day). The DSD function had a similar shape to that of the egrets, showing a peak in predicted cell use frequency at ~500 DSD, an effect strengthened in shallow water. Radial spatial (x,y) smoothing was applied to the landscape, but no additional differences were found among regions.

Cell use by storks was best predicted by the terms Depth, Depth^2^, Recession^2^, DSD, DSD^2^, HP, HP^2^, and Depth* DSD (Table 6). The form of the depth response was similar to that of ibis with a peak at low values of depth (~15 cm). Quadratic DSD and hydroperiod terms showed a peak in use at ~450 DSD, a sharp decline thereafter, and an increase in use at longer hydroperiods. Finally, differences in DSD primarily affected use of shallow water, with use increasing under long DSD (> 343 days) and decreasing under short DSD (< 343 days). This corroborates results from the egret and ibis model that higher DSD use was associated with many more birds when the associated high prey density was concentrated in shallow water. After accounting for residual spatial correlation, cell use by storks was higher in ENP and slightly lower in BCNP.

## Discussion

### Dynamic Habitat Selection

Results confirmed that selection for individual resources (prey availability, concentration, and production) varied given their availability across a decadal gradient of environmental conditions (i.e., dynamic habitat selection). Selection was further influenced by the availability of resources produced over differing temporal scales (daily to multi-annual). This often occurred under low resource availability, but also under circumstances when resources were plentiful and food could be selectively exploited.

We restricted habitat availability (i.e., depths) to observed wading bird foraging ranges, and all species selected locations with shallower water when available landscape depths were at the high and low ends of the hydrological gradient. In the early dry season when most of the landscape is wet, the few shallow areas are targeted by wading birds. In contrast, at the end the dry season, birds select shallower water to exploit concentrated prey resources. These results suggest that selection for water depth can occur in response to both limitation and exploitation over the gradient of landscape conditions. This idea is supported by wading bird resource selection functions, demonstrating that selection of water depths can change with different available conditions [17].

In addition, egrets and ibises selected for shallower water when the drying rate was high, but this was only observed in ibises when areas with a long prior wet period were available. This behavior in ibis could reflect their tendency to switch from feeding on crayfish at relatively higher landscape water depths to fish when they become highly concentrated under dryer conditions [43]. Egrets have a broader depth tolerance, but will opportunistically feed in shallow water to access concentrated fish that high recession rates provide [13,17]. In contrast to egret and ibises, the storks’ use of shallow water was not associated with recession rates, but instead with longer hydrological inundation. This behavior would allow storks to target larger sizes of marsh fishes that develop in deeper pools that may not dry annually [44].

All species used higher recession rates when available, further demonstrating its important function as a prey concentrating process. Selection of higher rates was most evident when recession rate heterogeneity was high, driven by local rainfall events. These temporary interruptions to the drying process can lead to prey dispersal and reduced rates of capture [13,16]. However, this study demonstrates that wading birds can identify locations where disruptions have been minimal. Higher recession rates were also selected in a dryer landscape when these sites could be accessed in shallow water. Similar to depth use, selection occurred when a particular resource was potentially limiting and when it could be exploited.

Few studies have directly modeled wading bird habitat selection influenced by multi-annual hydrological patterns; however, the population dynamics of fishes at this scale are well documented [33,45–46]. A higher frequency of severe drydowns (i.e., shorter DSD) limits the population of large fishes, whereas some species of small fish rapidly recolonize and crayfish emerge from burrows in newly flooded areas. This likely causes small variations in wading bird species’ responses to the length of the inundation period based on preferences of prey species. Thus, the length a site has been wet is only relevant to birds when it enters and remains in the species’ depth range and was modeled accordingly. Similar to recession rate use, all species used longer DSD when available, demonstrating its utility in describing long-term prey dynamics.

In a previous study, egrets and ibises selected sites with a longer wet period in a year with high prey availability, but not in years when prey was limiting [17]. Egrets and ibises in the current study selected for longer DSD when mean available landscape depths were greater than 15 and 10 cm, respectively; however, this selection decreased as their selection of shallower water increased. This demonstrates the importance of antecedent conditions during the wet season and as a prerequisite for efficient foraging (when combined with steady recession) later in the dry season. In contrast, DSD selection by storks did not vary with landscape conditions because they still used long DSD in shallow water and did not select longer DSD in wet conditions. All species also used sites with a longer wet period by selecting locations with greater depths, a response that likely benefits the species with a broader depth tolerance allowing them to forage in sites with a more abundant and diverse prey base [33,45] with larger body sizes [46]. For egrets, selection for a long wet period slightly increased as recession rates slowed, which could indicate their ability to locate higher density sites when prey is not being further concentrated by the drying process.

### Abundance over Environmental Gradients

Similar to resource selection patterns, temporal models of both flock and individual abundance indicate that wading birds are responding to and may be limited by processes interacting over the multiple spatio-temporal scales that influence prey availability. For example, for all species, the largest numbers of foraging birds were observed when the mean DSD used was ~400–600 days, consistent with the idea that long-term wetland inundation (across a gradient of hydroperiods) allows for prey production. Even under these conditions, however, the timing of greatest numbers of foraging birds differed among species depending on the availability and selection of suitable depths to access the increased prey production. Egrets were most abundant when locations with a long DSD (~500 days) were used and individuals fed in deeper water (15–25 cm), but egret abundance declined under the driest landscape conditions observed (< 15 cm mean availability). Ibises were also more abundant when they used longer DSD (~450 days), but the increase in abundance was instead strengthened when ibises used shallower water (10–20 cm). Indeed, in a year with high prey production and high nesting success, collected boluses of ibises predominately contained small fish that were concentrated in shallow water [43,47]. Abundance of storks did not directly respond to changing selection for DSD, but rather their abundance was highest in a dry landscape when individuals fed in shallow water (0–10 cm). The increased use of shallower water in a high production (i.e., DSD) environment demonstrates the fundamental importance of an established multi-annual prey base for all species. These conditions would occur when the majority of foraging habitat did not dry in the prior year.

Peak flock and individual abundance were observed in all species when landscape depth heterogeneity was high and individuals had a variety of depth choices. Under the wettest conditions, high heterogeneity was available to egrets, which can exploit deeper water. For ibis and storks, high depth heterogeneity occurred at the middle one-third of the hydrological gradient and limited ibis and stork abundance under wetter (and dryer) conditions. This reaffirms the importance of a landscape high in spatial heterogeneity from previous reports [13,17,25] and demonstrates that the significant loss of short hydroperiod wetlands may have caused a delay in nesting initiations of searcher species by decreasing the depth heterogeneity at the beginning of particularly wet years [13,25]. This spatial limitation also increases wading bird dependence on high drying rates to provide shallow depths early in the dry season that may have changed the species response to recession rate. While high recession rates were not necessarily a historical requirement of successful breeding, they provide new high quality patches that are exposed in suitable depths and may ease the loss of foraging habitat that would have been suitable under wetter conditions [17,26].

In this study, egret abundance was higher with the use of intermediate recession rates in a form similar to a model predicting their daily nest survival [24], with a peak in abundance at ~0.5 cm/day. Stork abundance was similarly higher with the use of intermediate recession rates. For these species, other studies have demonstrated a positive linear response between abundance or use and increasing recession rate [16–17,48], although there are several examples when recession rate does not contribute to increased probability of use [17,49], demonstrating that the species response to recession rate varies given available landscape conditions. In a year with high food availability, both egrets and storks did not demonstrate selection for recession rate illustrating that selection may occur specifically in less than optimal conditions [17,49] and may be a misleading model for habitat quality without the context of processes occurring at longer temporal scales.

### Conspecific Attraction

For all species, changes in resource levels caused individual abundance to rise more rapidly than flock abundance. For example, egrets clustered within EDEN cells as the landscape dried, indicated by a negative response of flocks but not individuals to greater depth. Individuals cluster into larger flocks when the benefits of conspecific presence outweigh the costs of interference [50]. This dichotomy provides a good indicator of when species with higher costs of interference use high quality habitat. As visual foragers, egrets incur higher interference costs when foraging in groups; however they clustered when food was plentiful as species converged on the most profitable prey resources [13,17]. Ibis dispersed following dry season reversals, and storks clustered at intermediate depths, demonstrating that social attraction can co-vary with foraging strategy, water-level changes, and habitat quality. As searchers and tactile foragers, ibises and storks are less sensitive to interference and maintained a higher level of conspecific attraction regardless of habitat conditions, likely resulting from specialized foraging strategies where conspecific presence helps reduce search and travel time [21,49,51]. Therefore, flock abundance in ibises and storks is more likely to indicate habitat quality and represent a larger area of the landscape suitable for foraging [14].

### Accounting for Spatial Autocorrelation

Typical of hydrological surfaces, the values of variables sampled at nearby locations were not independent of each other, resulting in spatial autocorrelation [52]. Accordingly, high residual spatial correlation in wading bird foraging distributions was substantially reduced by applying a non-parametric radial smoother, indicating spatial covariates not captured by the environmental variables sampled [40]. While the hydrological variables in this study are strongly linked with wading bird habitat use, other spatially correlated variables, not available in this study (e.g., nutrients, topography and vegetation structure) can influence prey availability [13,17]. Topography could play a particularly important role in accounting for spatial autocorrelation at the landscape scale, because large areas of wetland could drain into particular cells and highly concentrate fish leading to frequent occurrences of birds over a decadal scale.

While it has been demonstrated that environmental relationships can invert when accounting for spatial autocorrelation [53], the results from this study confirm spatio-temporal interactions identified by the TFC model. Again, the co-occurrence of cells with long DSD (> 400 days) and shallow water was associated with more frequent use by all species. Conversely, frequency of use of a cell declined in shallow water when DSD (and therefore time for prey reproduction) was short, indicating that birds may use these locations less even with optimal depths. If applied on an annual scale, this could indicate longer site fidelity in more resource dense cells, with birds potentially relying less on recession to expose new patches and less habitat overall over the length of the breeding season. In addition, egrets and storks used cells with a longer hydroperiod more frequently, demonstrating their energetic demand for larger and more abundant prey that could be exploited over repeated foraging bouts to the same location. This study also supports the idea that egrets select higher recession rates (which concentrate prey) to make up for low overall prey density and size. While a similar selection response was noted in ibises [17], this behavior did not increase frequency of use over the gradient of habitat conditions observed in the current study. The behavior was only demonstrated (and may only be effective) in a particularly dry year, when ibis switch to concentrated fish as a prey resource [43].

Similar to the results of the TFC model, a sharper decline was evident in frequency of cell use by ibis and storks when DSD exceeded 600 days; however, use of these cells tended to co-occur with the use of deeper water. This could demonstrate the searchers’ higher giving up density (GUD) in these depths [13]. Alternatively, the species’ response could have decreased because of changes in the prey community as a result of long periods of inundation. Under these habitat conditions, patches become dominated by larger predatory fishes and limit crayfish populations [54]. Periodic droughts may benefit ibis indirectly by increasing crayfish populations, potentially driving the large pulses in ibis nesting effort that follow drought years [55].

### Application

Our findings provide context to species-specific resource selection and foraging distributions of wading birds and are consistent with other patterns observed, but offer a better model for understanding how recent trends in water levels and spatial limitations in the availability of prey have driven species-specific population trends. Results can also be applied to evaluate the multi-annual resource pulses of real-time conditions, climate change scenarios, or restorative hydrological regimes by tracking changing seasonal and annual locations of high quality foraging patches. While these specific models (Wading Bird Distribution Evaluation Models; WADEM) are limited to the Greater Everglades, the modeling framework could easily be applied to other dynamic ecosystems with long temporal records of species distribution data. Our method of aggregating data and isolating spatial and temporal scales into distinct modeling components can provide a clearer picture of species responses across resource gradients, particularly when the focus is on species responses to dynamic variables, such as water levels.

The TFC predicts the daily abundance of flocks and individuals on the landscape within a changing area of suitable depths throughout the nesting season of each species. The model is updated every day based on the average response to resource levels within suitable habitat (i.e., depths) while simultaneously incorporating the response to resource heterogeneity. Hence, the changing preference for resource levels is captured and evaluated by the landscape response. Because only resources within suitable habitat were modeled, the response can represent patch quality, summed over the extent of the Everglades. The SFC predicts spatial occurrence for each cell by integrating spatial dynamics unaccounted for by the chosen set of predictors (i.e., spatial correlation). When this model is averaged spatially over daily time steps, it can serve as a surrogate measure of patch abundance. Finally, combining daily output from the SFC and TFC models can jointly account for the seasonal increase and eventual decrease in patch abundance as the landscape dries, and the increase in patch quality as habitat with longer periods of inundation becomes available in suitable water depths.

In the Everglades, wading birds are intimately connected with rapidly changing hydrology and with each other, thus serving as indicators of ecosystem health and restoration efforts mandated by the Water Resources Development Act [56]. Ecological models that incorporate species’ ecology such as dynamic habitat selection, conspecific attraction, and abundance over environmental gradients provide more realistic predictions to facilitate restoration success. Further, evaluation of these models using metrics of reproductive success (e.g., nesting effort, nesting success) has provided a direct measure of habitat quality to compare alternative restoration plans [57].
